# Properties of collagen-based hemostatic patch compared to oxidized cellulose-based patch

**DOI:** 10.1007/s10856-018-6078-9

**Published:** 2018-05-23

**Authors:** Paul Slezak, Xavier Monforte, James Ferguson, Sanja Sutalo, Heinz Redl, Heinz Gulle, Daniel Spazierer

**Affiliations:** 1 Ludwig Boltzmann Institute for Experimental and Clinical Traumatology, Donaueschingenstrasse 13, A-1200 Vienna, Austria; 2 Baxter Medical Products GmbH, Stella-Klein-Loew Weg 15, A-1020 Vienna, Austria

## Abstract

Two self-adhering hemostatic patches, based on either PEG-coated collagen (PCC) or PEG-coated oxidized cellulose (PCOC), are compared regarding to maximum burst pressure, mechanical stability, and swelling. In addition, the induction of tissue adhesions by the materials was assessed in a rabbit liver abrasion model. Both materials showed comparable sealing efficacy in a burst pressure test (37 ± 16 vs. 35 ± 8 mmHg, *P* = 0.730). After incubation in human plasma, PCC retained its mechanical properties over the test period of 8 h, while PCOC showed faster degradation after the 2 h time-point. The degradation led to a significantly decreased force at break (minimum force at break 0.55 N during 8 h for PCC, 0.27 N for PCOC; *p* < 0.001). Further, PCC allowed significantly higher deformation before break (52% after 4 h and 50% after 8 h for PCC, 18% after 4 h and 23% after 8 h for PCOC; *p* = 0.003 and *p* < 0.001 for 4 h and 8 h, respectively) and showed less swelling in human plasma (maximum increase in thickness: ~20% PCC, ~100% PCOC). Faster degradation of PCOC was visible macroscopically and histologically in vivo after 14 days. PCC showed visible structural residues with little cellular infiltration while strong infiltration with no remaining structural material was seen with PCOC. In vivo, a higher incidence of adhesion formation after PCOC application was detected. In conclusion, PCC has more reliable mechanical properties, reduced swelling, and less adhesion formation than PCOC. PCC may offer greater clinical benefit for surgeons in procedures that have potential risk for body fluid leakage or that require prolonged mechanical stability.

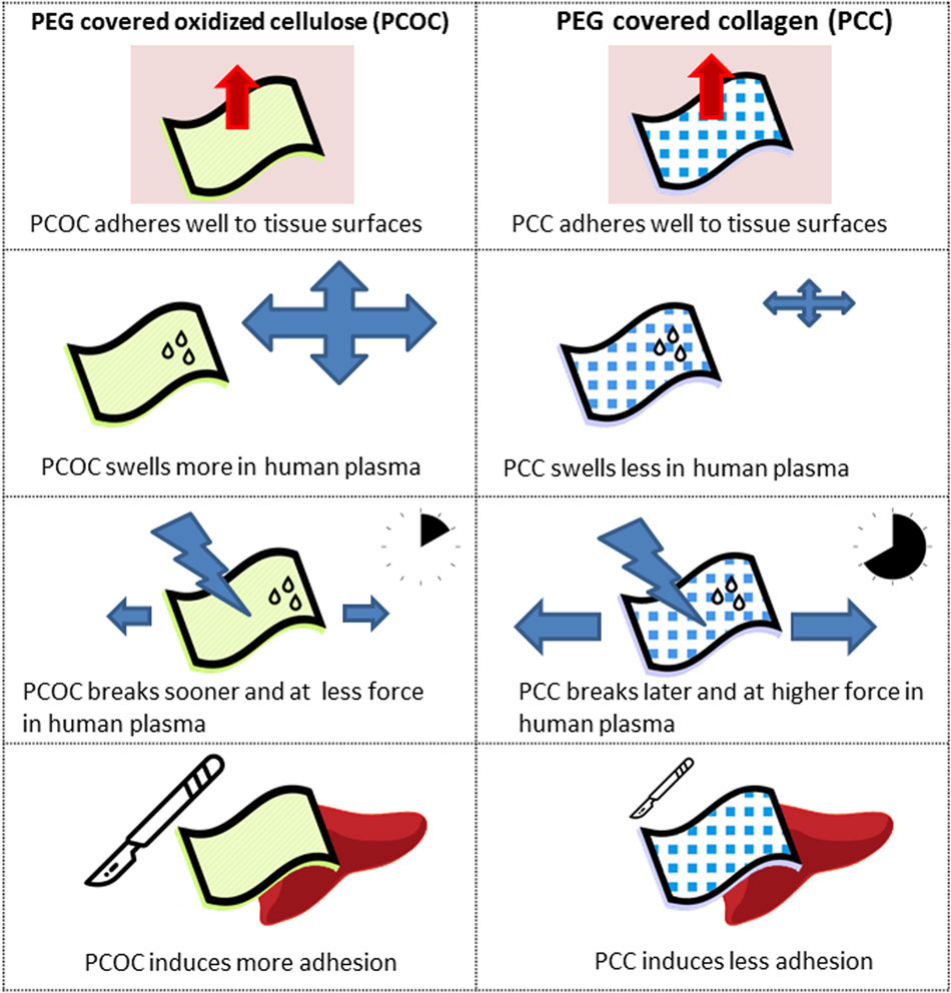

## Background

Along with the surgical control of bleeding with sutures and electrocautery, the use of local hemostats is the current standard of care [1]. There are several classes of hemostats: liquids (e.g., fibrin sealants), powders (e.g., starch particles), flowables (e.g., gelatin particles with thrombin [2]) and patches (e.g., fibrinogen and thrombin coated collagen). In order to choose the most appropriate hemostat for the clinical situation, it is essential to understand the mechanism of action, efficacy, and possible adverse events of each material [3].

The first hemostatic patches were based upon collagen [4], gelatin [4] or oxycellulose [4, 5] without additional coating, but were partly used in conjunction with (fibrin) sealants [4]. Later on, improved devices were coated with fibrinogen and thrombin to improve their hemostatic performance and to provide tissue sealing [6]. These hemostatic pads consisted of a sheet-like backing and a self-adhering surface. More recently developed hemostats are utilizing collagen, neutralized oxidized cellulose, or an oxidized cellulose–polyglactin 910 composite backing. Fibrinogen and thrombin and synthetic or protein-reactive sealant components are used as the adhering surface. New hemostatic pads like polyethylenglycol (PEG)-coated collagen (PCC) [7, 8] and PEG-coated oxidized cellulose (PCOC) [9] have recently been developed and are used in surgical practice.

PCC is composed of a collagen backing coated with a synthetic N-hydroxysuccinimide functionalized polyethylene glycol (NHS-PEG), which rapidly affixes the collagen pad to tissue [10]. Collagen induces clot formation through platelet activation and NHS-PEG covalently binds the pad to the tissue surface and seals the wound effectively even when hemostasis is impaired by heparinization and antiplatelet therapy [11].

PCOC is comprised of an absorbable backing made of neutralized oxidized cellulose and self-adhesive hydrogel components. The neutralized oxidized cellulose absorbs the blood, while the hydrogel creates a barrier for blood and adheres the patch to the bleeding site [12].

Existing evidence suggests that hemostatic patches enable delivery of pro-coagulants to defined areas with less chance of dilution and/or displacement by blood flow, but they require a pressure buttress for a suitable amount of time to achieve good results [13]. In addition, some patches have effective sealing properties, as well as hemostatic capabilities and as such can be used as a clinical sealant. There are several surgical applications where sealing of tissues is of high clinical importance: e.g., preventing blood loss from vascular reconstructions, preventing air leakage from a pulmonary reconstruction, and preventing cerebrospinal fluid (CSF) loss from a durotomy. Across these wide clinical applications, a patch with a high elasticity and material integrity throughout the postoperative period is critical to ensure hemostasis or sealing efficacy.

Another consideration is that the foreign material may be a stage for postoperative adhesion formation, which can lead to significant postoperative morbidity for the patient e.g., in cardiac procedures with pericardial adhesion formation [14] or in abdominal surgery after anastomosis or intraperitoneal hernia repair [15]. In this study, we compared two self-adhering hemostatic patches in vitro with regard to maximum burst pressure, mechanical stability, and swelling over time in wet state as well as *in vivo* with regard to adhesion formation.

## Materials and methods

### Self-adhering hemostatic patches

#### PEG-coated collagen patch (PCC)–Hemopatch

Hemopatch (Sealing Hemostat, Baxter AG, Vienna, Austria) consists of a soft, thin, pliable, flexible pad of collagen derived from bovine dermis, coated with NHS-PEG (pentaerythritol polyethylene glycol ether tetra-succinimidyl glutarate). Is intended as a hemostatic device and surgical sealant for procedures in which control of bleeding or leakage of other body fluids or air by conventional surgical techniques is either ineffective or impractical.

#### PEG-coated oxidized cellulose patch (PCOC)–Veriset

Veriset (Hemostatic Patch, Covidien llc, Mansfield, USA) is comprised of oxidized cellulose impregnated with buffer salts, trilysine and a reactive polyethylene glycol (PEG). It is intended for use in solid organ and soft tissue procedures as an adjunct to hemostasis when the control of capillary, venous, arteriolar bleeding by pressure, ligature or other conventional methods is ineffective or impractical.

### Burst pressure

Burst pressure was measured *in vitro* using a previously reported test model [16]. Briefly, circular test specimens with a diameter of 25 mm (*n* = 15 per group) were applied to a punctured (4 mm circular defect) collagen film (Nippi incorporated, Tokyo, Japan) in the presence of 200 µl of re-calcified citrated human blood, effectively sealing it, mimicking a clinical situation at the tissue to product interface. Test items were approximated for 2 min by placing a dry gauze and a 200 g weight on top of the sample with a 37 °C warm heating pad below.

The test system consisted of a pressure barrel onto which the sealed collagen film was mounted. Hydrostatic pressure was increased below the collagen film by infusion of citrated blood (50 ml/h) using an infusion pump (Perfusor fm, Braun, Germany). Fluid pressure and sealing efficacy of the collagen film were monitored. The recorded pressure at product failure was defined as the burst pressure.

The test system was selected because it allows comparison of substrate, adhesive and cohesive strength, and it simulates the sealing of tissue surfaces under worst possible conditions.

### Mechanical stability

Both test items were cut into dog bone shape for mechanical testing with a central region of 1 cm width and 2 cm length (6 replicates). When a specimen did not break within the defined central area the measurement was considered invalid. The specimens were tested after incubation in thawed human citrated plasma at 37 °C either for 30 min, 2, 4, or 8 h.

After incubation, the specimens were mounted on a uniaxial testing system (Zwick/Roell type BZ2.5/TN1S) with a 50 N load cell type B066120.03.00, pre-loaded with 0.01 N, and subsequently strained until failure at a constant speed of 20 mm/min. Changes in the force at break and deformation at break were recorded.

### Swelling kinetics

Patches (2.5 × 2.5 cm) of both test items were submerged in thawed human plasma at room temperature. No manual pressure was applied before submersion. The mean swelling volume and the mean thickness were plotted against time. The investigated time points were 0 h, 30 min, 4, 8, 24, 48 and 72 h (*N* = 5/group/time point). The plasma uptake of the specimens was determined by measuring the weight on an analytical scale before and after swelling.

The volume of the specimens was determined by measuring their length, width and thickness. The thickness was measured with an electronic caliper at 5 evenly distributed positions across each patch. In order to flatten wrinkles and uneven surfaces, slight manual pressure was exerted until the caliper’s sensor was evenly approximated to the surface. A rectangular sensor tip with a size of 1 × 1 cm was used to obtain standardized approximation.

The area of the specimens was measured planimetrically from digital images obtained from a position perpendicular to the specimens, minimizing perspective distortion. The images featured a metric scale and were analyzed using the planimetric software “LUCIA G” version 4.80.

### Rabbit liver abrasion model adhesion formation

This study was performed according to the Austrian Ordinance on Animal Experiments: BGBl Nr. 501/1989. Approval to this study by the Animal Protocol Review Board of the City of Vienna was obtained prior to conducting experiments.

PCC and PCOC were compared in a rabbit liver abrasion model with regard to adhesion formation 2 weeks after product application (*N* = 9/group). 18 male New Zealand White rabbits weighing 2.35–3.64 kg were used. A celiotomy was performed and the left lobe of the liver exposed. A superficial circular lesion of about 1.3 cm in diameter was created on the liver with a scalpel and/or scissors to a depth of approximately 2 mm. 3 × 3 cm patches were used to cover the defects.

The wound was swabbed with dry gauze before application of the test items, which was performed according to the respective IFU. PCC was applied and approximated with dry gauze for 2 min using mild digital pressure. PCOC was applied and approximated for 30 s, after which the lesion was visually inspected. In the case of sustained bleeding it was approximated for an additional 30 s.

The liver was then returned to its original position, the omentum resected and the abdominal wall closed with everting horizontal mattress sutures. Following surgery, rabbits were given Buprenophine (0.05 mg/kg) every 8 h for 4 days subcutaneously as a postoperative analgesic.

After 14 days, animals were humanely euthanized under deep anesthesia by an overdose of thiopental sodium. The abdomen was visually inspected for pathological findings, and the presence and severity of adhesions were rated (Table 1) [17, 18].

**Table 1:** Adhesion formation size and severity grades

Adhesion sizes:	Size 0 No adhesion	Size 1 1–25% patch size	Size 2 26–50% patch size	Size 3 51–75% patch size
Adhesion severity:	No adhesion	Grade 1 Filmy adhesion, blunt dissection possible	Grade 2 Strong adhesion, sharp dissection necessary	Grade 3 Very strong, vascularized adhesion, sharp dissection, damage to tissue not preventable.

### Statistics

All values reported are mean values followed by the standard deviation (SD). All graphs show mean ± SD. Results of the 2 groups (PCC vs. PCOC) were statistically compared to each other using a two sample *t*-test, and considered significant when *p* < 0.05.

## Results

### Burst pressure

**Fig. 1:**
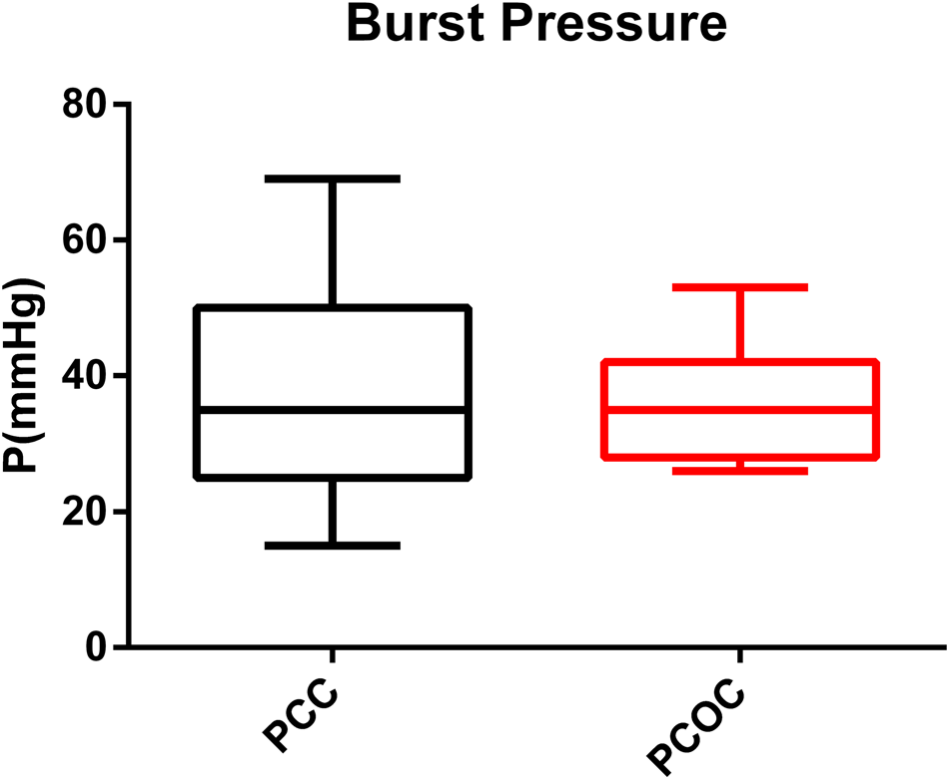
PCC and PCOC had similar mean burst pressure in an *in vitro* burst pressure system (*p* = 0.730)

The burst pressure values were 37 ± 16 mmHg (*N* = 15) for PCC and 35 ± 8 mmHg (*N* = 15) for PCOC [Fig. 1]. No significant difference between these two groups was detected (*p* = 0.730).

### Mechanical testing

**Fig. 2:**
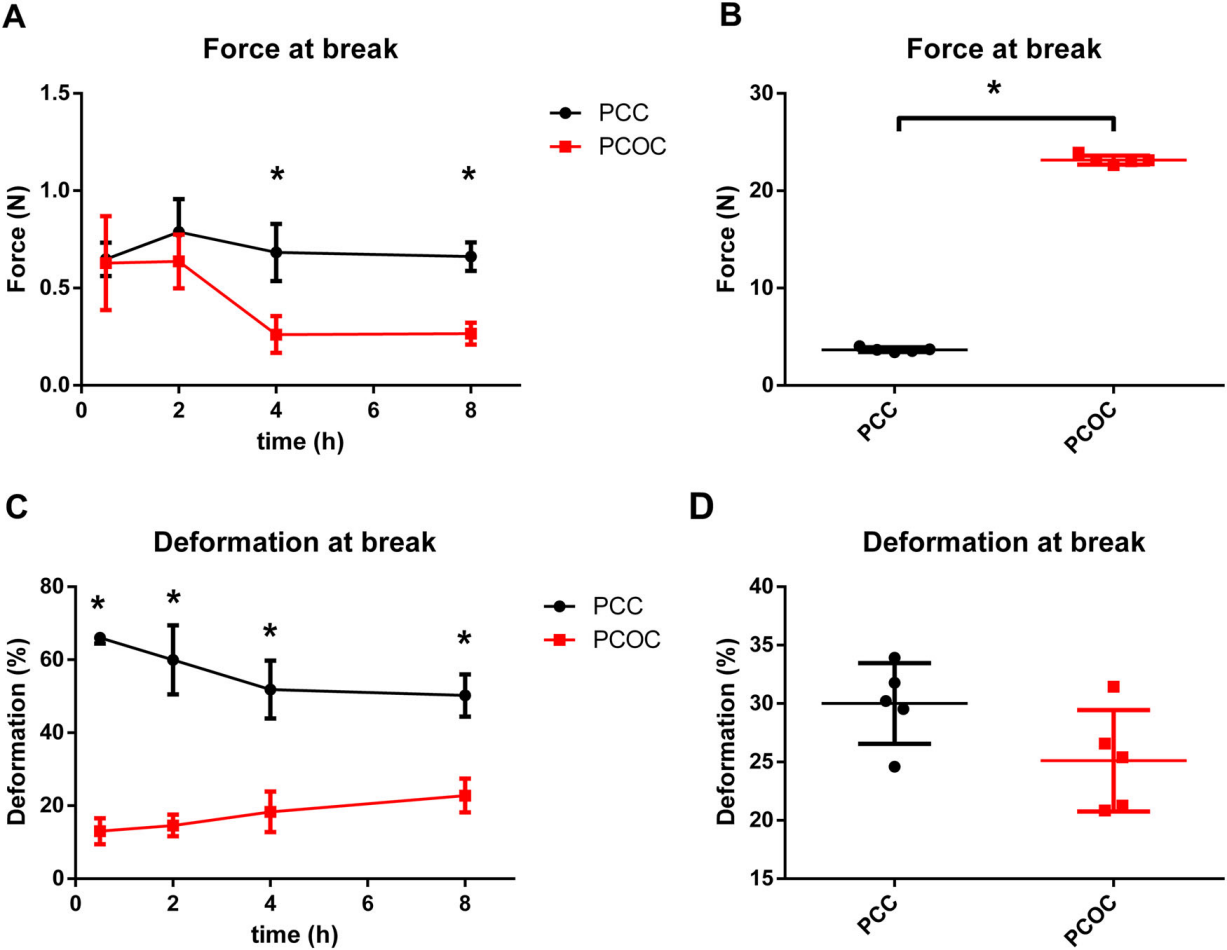
Hemopatch (PCC) provides greater mechanical strength over time than Veriset (PCOC). Force at break (**a**, **b**) and deformation at break (**c**, **d**) were measured after incubation of specimens in thawed human plasma for 30 min, 2, 4 and 8 h (**a**, **c**) as well as of dry materials (**b**, **d**). Note that for all experiments *n* = 6, however only valid measurements are included in the data analysis: Veriset at 4 h (*n* = 2) and Veriset at 8 h (*n* = 3), all other timepoints and samples (*n* = 5). Significant different time points (*p* < 0.05) are marked with an asterisk

The mean force at break of plasma incubated PCC was approximately 0.7 N over the entire test period of 8 h [Fig. 2a]. PCOC performed comparably within the first 2 h (*p* = 0.869 and 0.160 for 30 min and 2 h, respectively) but the force at break decreased significantly thereafter, reaching 0.26 N (41% of the 2 h value) after 4 h of incubation in human plasma (*p* = 0.015). The mean force at break for dry PCC was 3.67 ± 0.24 N [Fig. 2b]. For dry PCOC the mean was significantly higher with 23.16 ± 0.47 N (*p* < 0.001).

The deformation at break of PCC specimens was stable at approximately 50% over the 8 h incubation period [Fig. 2c]. The values for PCOC were significantly lower (i.e., 13% by 30 min; *p* < 0.001). Deformation of PCOC increased at 4 and 8 h but was still significantly smaller than of PCC (18% after 4 h and 23% after 8 h for PCOC; 52% after 4 h and 50% after 8 h for PCC; *p* = 0.003 and *p* < 0.001 for 4 and 8 h, respectively). The mean deformation at break of dry PCC was 30.01% ± 3.47 and of dry PCOC 25.11% ± 4.35 [Fig. 2d; *p* = 0.084].

Notably, only a limited number of PCOC specimens were tested successfully after the 2 h time point, as either failure occurred outside of the defined central test area (invalid sample), or proper handling of the specimen was not possible due to advanced degradation. This resulted in a sample size of *n* = 2 at 4 h and *n* = 3 at 8 h for PCOC.

### Swelling in plasma

**Fig. 3:**
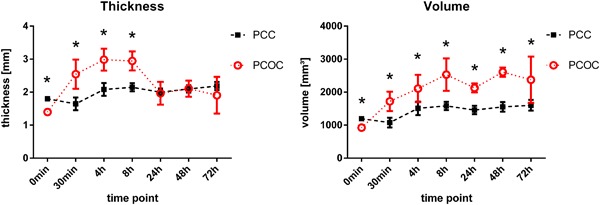
Hemopatch (PCC) swells less than Veriset (PCOC). Both products showed an increase in thickness and volume during the first 4–8 h in human plasma. The increase in both thickness and volume for Hemopatch was moderate and substantially higher for Veriset, which showed a sharp drop in thickness at later time points. Significant different time points (*p* < 0.05) are marked with an asterisk

**Fig. 4:**
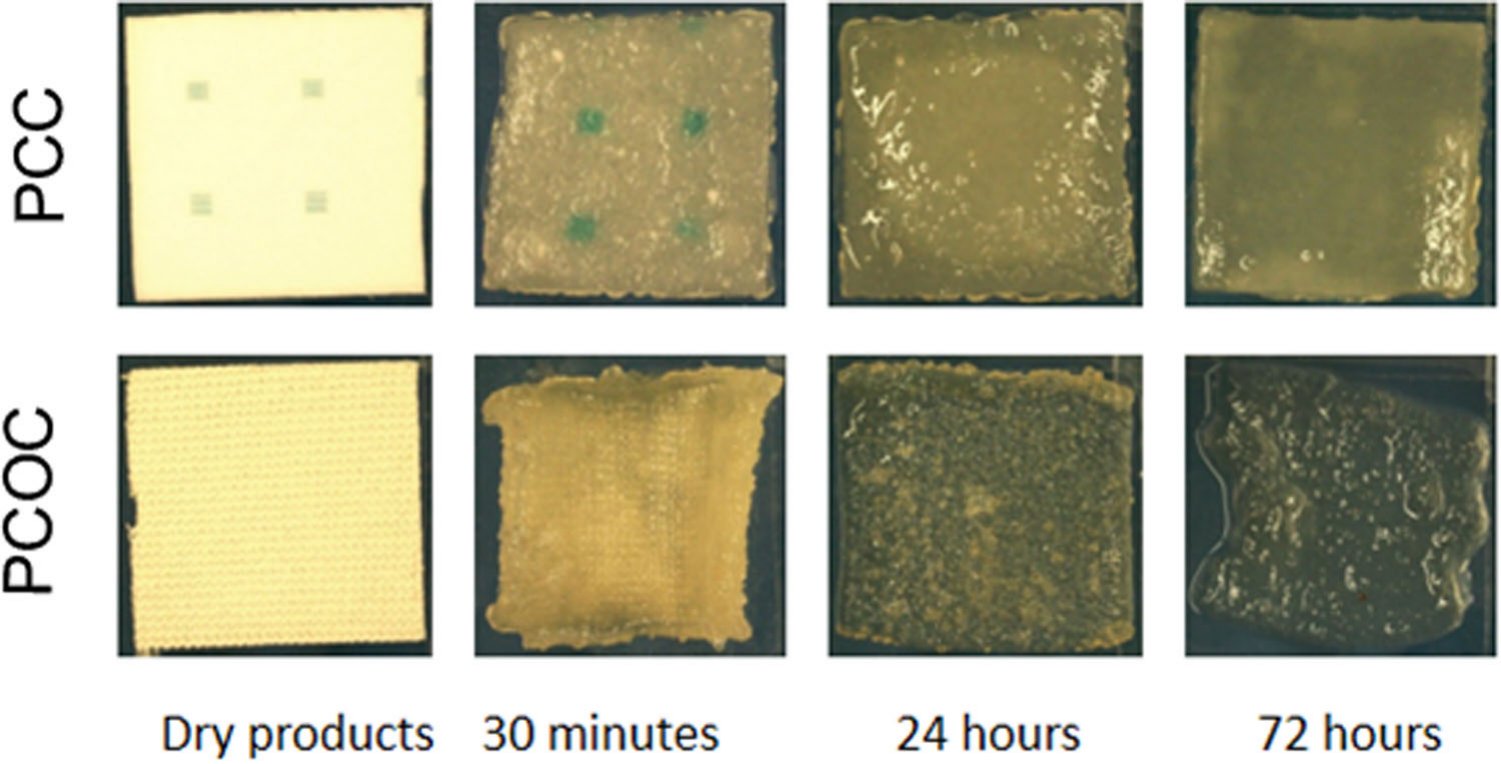
Veriset (PCOC) has a rapid breakdown in plasma relative to Hemopatch (PCC). Both products were incubated for a period of 72 h in thawed human plasma during which rapid degradation of Veriset was observed. After 30 min, Veriset showed strong curling of the edges which became less over time as the mechanical stability declined. The manual handling of Veriset samples was difficult at late time points, i.e., 48 and 72 h due to mechanical instability. Hemopatch was much more stable, did not show any curling, and handling was easy during the entire duration of the experiment

In plasma, PCC reached the maximum volume and thickness after 4 h with no appreciable changes observed thereafter. PCOC reached the maximum volume after 4 h and the maximum thickness after 30 min [Fig. 3]. The volume did not change thereafter, however, the thickness of PCOC decreased considerably after 8 h, accompanied by disintegration of the material [Fig. 4]. PCOC increased approximately 100% in thickness as compared to the dry value, while PCC showed an increase of approximately 20%, indicating a significantly lower swelling compared to PCOC (*p* < 0.010 at all time-points up to  8 h). In absolute numbers the thickness of PCOC after swelling was approximately 3 mm and of PCC approximately 2.2 mm. In terms of volume change, PCC increased up to 34% of its dry volume (72 h), while PCOC increased up to 182% (48 h), showing a statistically significant difference (*p* < 0.050) at all time-points.

### In vivo adhesion formation

**Fig. 5:**
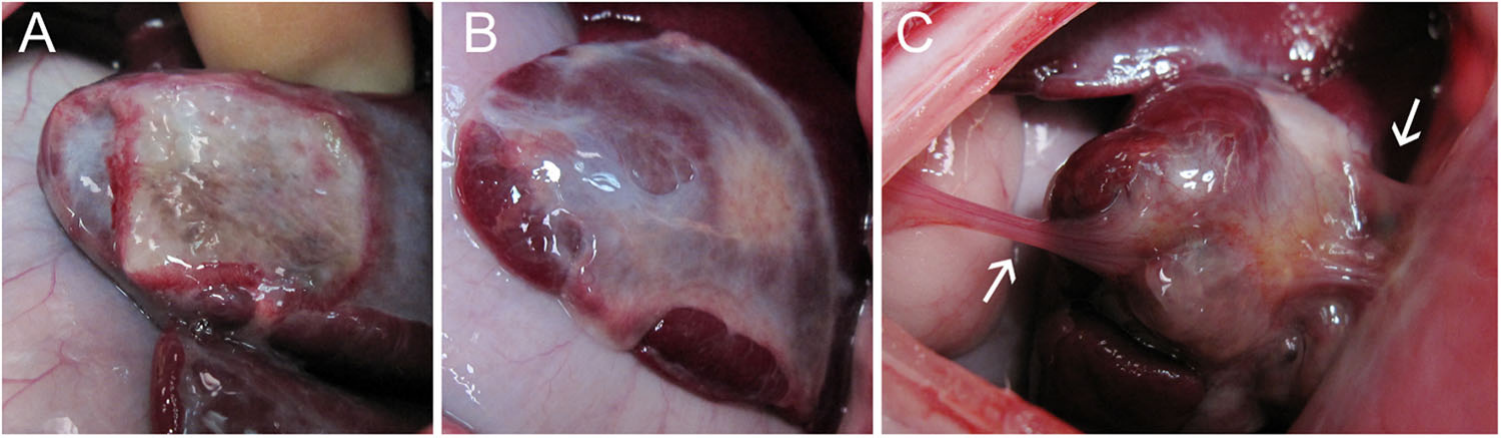
Veriset (PCOC) degraded faster in vivo and showed increased adhesion formation than Hemopatch (PCC). Representative images of remaining material of Hemopatch (**a**), and Veriset without (**b**) and with adhesion (**c**) in a rabbit liver abrasion model 14 days after implantation. In the Veriset group, adhesions (marked by arrows) were detected in 4 out of 9 animals

**Fig. 6:**
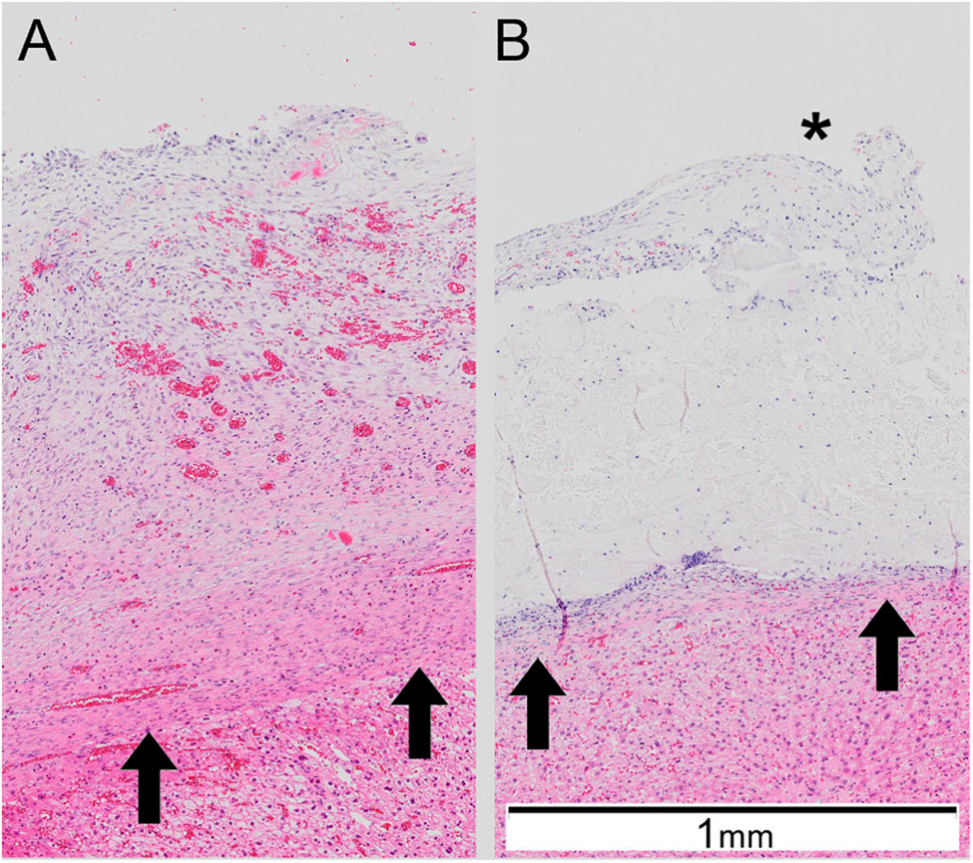
Representative hematoxylin and eosin stained images of Veriset (**a**) and Hemopatch (**b)** and surrounding tissue. Hemopatch shows clearly visible structured material residues with little cellular infiltration, and connective tissue coverage (marked with asterisk (*)). Veriset shows strong cellular infiltration and vascularization with no structured material residues visible. The interface of the liver to the hemostatic material is marked with arrows

In the PCOC group 4 out of 9 animals showed adhesion formation (Table 2). In the PCC group 0 out of 9 showed adhesion formation. The observed adhesions were of medium size and grade (sizes 2–3, grade 2; Table 2, Fig. 5c). Initial hemostasis with both patches was good with no bleedings detected 2 min after application (hemostasis data not shown). Macroscopically, PCC showed less degradation compared to PCOC after 14 days (Fig. 5), which was also confirmed by histology (Fig. 6). A considerable amount of structured PCC material was still visible, which was covered by a layer of connective tissue. Hematoxylin and Eosin staining revealed almost no cell infiltration within the remaining PCC material. In contrast, PCOC showed strong cellular infiltration and vascularization throughout the remaining patch with no structured material residues visible (Fig.6).

**Table 2:** Hemopatch (PCC) was less adhesiogenic than Veriset (PCOC) in a rabbit model

Adhesion	Size 0 No adhesion	Size 1 1–25% of patch size	Size 2 26–50% of patch size	Size 3 51–75% of patch size	Size 4 76–100% of patch size
**PCC**	9	0	0	0	0
**PCOC**	5	0	1 (grade 2)	3 (grade 2)	0

Either Hemopatch or Veriset was applied on a rabbit liver abrasion. Two weeks later adhesion formation was assessed. Grade 0 = no adhesion, 1 = filmy adhesion, blunt dissection possible, 2 = strong adhesion, sharp dissection necessary, 3 = very strong, vascularized adhesion, sharp dissection, damage to tissue not preventable.

## Discussion

Hemostatic patches are used in a variety of clinical indications [1], however their intrinsic material properties determine which product is best suited for a specific application. Surgeries with potential leakage of body fluids like CSF [19], bile or urine require efficient sealing, while different surgical application sites all have their own specific requirements on material properties. Spinal surgery for instance requires a minimum swelling behavior [20], anastomotic sealing following esophageal surgery [21] or lung applications [16] benefit from a certain elasticity of the used patches.

In this study, we evaluated the material properties of two commercially available patches that differ mainly in the biomaterial used as backing. PCOC, an oxidized cellulose patch covered with a reactive PEG, is indicated for hemostasis only. PCC, a reactive PEG-coated collagen patch, is indicated for hemostasis, sealing and for closure of dural defects. Our results show clear differences in the material properties based on the different biomaterials used, in particular for degradation and mechanical stability. Taking these results into consideration, both patches seem to be well suited for their intended use. Beyond these material characteristics, hemostatic material that remains in the body after surgery and gradually degrades should not induce adhesion formation between tissues at the site of application that could lead to adverse side effects. This concern was addressed in this study and while both of the tested hemostatic materials share a common principle and mode of action, the different backing materials influenced the outcome.

Burst pressure, an indicator for sealing efficacy was equally high for both products, which is likely due to the similar PEG-based chemistry creating the adhesive hydrogel. However, major differences in mechanical stability were detected over time. After 2 h of incubation in plasma, at time points that are still relevant clinically, a rapid decline in the mechanical strength of PCOC was observed. This behavior was linked to the rapid degradation of the oxidized cellulose backing in plasma. Even at the early time points of 30 min and 2 h, single samples of PCOC showed inferior mechanical properties with an already reduced force at break. In comparison, the collagen backing of PCC presented itself as a more suitable and reliable material for sealing indications, as it withstood higher stress and strain over a prolonged time. This early loss of mechanical stability of oxidized cellulose is assumably linked to the rapid degradation of its polyuronic acid component which is depolymerized by β-elimination that is facilitated by glycosidases [22], a process that takes place within hours, before the fibrous component is phagocytized. Collagen on the other hand is degraded much slower either by phagocytosis from surrounding macrophages or by secretion enzymes like matrix metalloproteinase [23]. This was confirmed by our histology assessment where considerable structural PCC material was present with only little cell infiltration after 14 days, while PCOC was fully infiltrated by cells and no structural material was detected anymore.

Prolonged sealing of tissues is of high clinical importance for example to protect patients from cerebrospinal fluid (CSF) loss after durotomy. In line with our data, the self-adhering PCC was successfully used as a dural substitute and proved effective as a dural sealant in this field [19]. A longer lasting material would also be preferred to prevent development of postoperative lung fistulas after lung surgery. Such air leaks remain a significant problem in thoracic surgery, especially if they persist for several postoperative days.

In addition to the longer lasting mechanical stability, PCC also allowed a higher deformation before mechanical failure during the first 2 h, making it the potentially superior material for hemostasis and sealing applications that require elasticity (e.g. lung sealing). As lung sealant, such a material will be beneficial to treat intraoperative alveolar air leaks which occur in a high number of patients during pulmonary resection despite routine use of sutures and stapling devices. In fact, a previously published study already suggested PCC to be efficacious intraoperatively in ventilated pig lungs upon partial resection [16].

With regard to patient safety, the swelling properties of hemostatic materials are a critical concern as they may compress vital structures when used in confined spaces [20]. Both of the tested materials did swell in human plasma, however the increase in thickness and in volume was much more pronounced with PCOC than with PCC.

Another major safety concern when using hemostats and sealants in clinical care is the incidence of adhesion formation after surgery. Since hemostatic patches remain within the body for a prolonged time after surgical intervention, both materials were tested in a relevant preclinical model with a follow up time of 2 weeks. In an article on adhesion prevention Robertson et al. [24] stated that oxidized regenerated cellulose (in its non-coated form) may increase the risk of adhesions if optimal hemostasis is not achieved. Along a similar line, González-Quintero and Cruz-Pachano [25] voiced concerns that an anti-adhesive oxidized cellulose barrier may aggravate rather than prevent adhesion formation in the presence of blood. To the best or our knowledge, no such reports are available for collagen.

In this study, we observed that PCOC was more prone to adhesion formation in our animal model (4 adhesions out of 9 applications) than PCC (0 adhesions out of 9 applications), despite its observed quicker degradation. A statistically powered study would be needed to confirm this finding.

## Conclusion

PCC showed superior mechanical properties to PCOC when exposed to human plasma mimicking in vivo conditions. PCC was more resistant to degradation than PCOC in vitro and in vivo, and retained its mechanical integrity over a longer time. PCC appears to have properties well suited for sealing and hemostasis in situations that require sustained structural stability and elasticity. In vivo, both products provided fast hemostasis in rabbit liver abrasions. However, our data indicate that PCOC leads to an increase in post-surgical adhesions in comparison to PCC.
